# Novel pH Selective, Highly Lytic Peptides Based on a Chimeric Influenza Hemagglutinin Peptide/Cell Penetrating Peptide Motif

**DOI:** 10.3390/molecules24112079

**Published:** 2019-05-31

**Authors:** Bethany Algayer, Ann O’Brien, Aaron Momose, Dennis J. Murphy, William Procopio, David M. Tellers, Thomas J. Tucker

**Affiliations:** Merck Research Laboratories, Merck and Co, Inc., West Point, PA 19486, USA; bethany_algayer@merck.com (B.A.); aobrien@incyte.com (A.O.); aam888010@icloud.com (A.M.); dennis.murphy@merck.com (D.J.M.); wmprocop@gmail.com (W.P.); david_tellers@merck.com (D.M.T.)

**Keywords:** peptides, endosomolytic, amphiphilic, fusogenic, influenza hemagglutinin, RBC lysis

## Abstract

Delivery of macromolecular cargos such as siRNA to the cytosol after endocytosis remains a critical challenge. Numerous approaches including viruses, lipid nanoparticles, polymeric constructs, and various peptide-based approaches have yet to yield a general solution to this delivery issue. In this manuscript, we describe our efforts to design novel endosomolytic peptides that could be used to facilitate the release of cargos from a late endosomal compartment. These amphiphilic peptides, based on a chimeric influenza hemagglutinin peptide/cell-penetrating peptide (CPP) template, utilize a pH-triggering mechanism in which the peptides are protonated after acidification of the endosome, and thereby adopt an alpha-helical conformation. The helical forms of the peptides are lytically active, while the non-protonated forms are much less or non-lytically active at physiological pH. Starting from an initial lead peptide (INF7-Tat), we systematically modified the sequence of the chimeric peptides to obtain peptides with greatly enhanced lytic activity that maintain good pH selectivity in a red blood cell hemolysis assay.

## 1. Introduction

The use of siRNA to treat various disorders remains a promising potential novel approach for future intervention [1]. Unfortunately, delivery remains a key unsolved issue. Following endocytosis, endosomal entrapment of siRNA-based cargos remains a critical unsolved barrier for efficient delivery of siRNA to the cytosol where it can engage RNA-induced silencing complex (RISC) and effect message knockdown [1,2]. Numerous approaches to enhancing endosomal escape have been tried or are currently under investigation [2], however to date none have solved the problem. Among these approaches, the use of cell penetrating (CPP) and/or endosomal escape peptides (EEP) has been featured in the literature [3]. The peptides have been used primarily as mixtures with siRNA [1], formulated into lipid nanoparticles (LNPs) [2], or in a few cases directly conjugated to the siRNA [4]. To date, none of these approaches has shown more than incremental improvement in cargo delivery.

As part of our investigation of siRNA delivery approaches, we decided to make use of small peptides as potential endosomal escape agents. Our initial strategy was to design novel, membrane lytic peptides that used a pH triggering mechanism as a potential safety margin. Similar approaches involving endolytic polymer scaffolds are also currently being investigated for the delivery of siRNA cargos [2]. Ideally, the small peptides would be delivered in a specific, targeted manner to the chosen target cell via conjugation to the cargo using an appropriate linker. After endocytosis, the peptide would be freed from the linker/cargo and concentrated in an endosome. Theoretically, as the endosome progressed through its life-cycle and the associated acidification process, one could take advantage of the pH drop to act as a trigger for the peptide. We hoped to design novel pH triggered peptides that were relatively inactive at physiological pH (7.4), but at late endosomal pH (5.5) were protonated and underwent a conformational change that conferred membrane lytic activity to the peptide. Such pH-triggered peptides are widely known in the literature and form the basis for the fusogenic peptides used by viruses to allow their genetic material to escape from endosomes and induce viral replication [5]. Since such viral processes are well evolved and quite efficient, we theorized that this would be an excellent mechanism to attempt to mimic for endosomal escape of siRNA-based cargos. Of course, the potential for toxicity would exist in such an endosomolytic process by releasing endosomal contents into the cytosol. Theoretically, any mechanism that results in the widespread leakage of endosomal contents into the cytoplasm could have this liability, and detailed studies to understand the limitations of these approaches and the potential for toxicity will clearly be needed. Despite this, it is possible that such a peptide might be useful for cytosolic delivery of endosomally-entrapped cargos, and such peptides have been studied in the literature and have in some cases been show to effect cargo delivery to the cytosol without inducing toxicity [6]. We were also interested in expanding our fundamental knowledge of the interaction of various classes of membrane active peptides with membranes, regardless of the potential for toxicity that is inherent in this mechanism. Extensive mechanistic studies in the literature with membrane active [7], lytic [8], fusogenic [9], and antimicrobial peptides [10] have demonstrated complex behavior and have shown diverse and often contradictory results based on the assay or system used to study the peptides. We wanted to avoid the complexity and diversity of these kinds of approaches, and instead chose to pursue an empirical, medicinal chemistry/structure-activity relationship approach to investigating and optimizing the lytic activity of this class of peptides. While numerous publications [11,12] have discussed the membrane disrupting properties of many peptides, there are no reports of highly systematic synthetic chemistry studies undertaken to optimize these peptides as potential endosomolytic agents. In this manuscript, we detail our efforts to design and systematically investigate the SAR of a series of novel, chimeric lytic peptides based on the influenza hemagglutinin peptide (HA2) and the well-characterized cell-penetrating/cargo delivery peptide Tat.

## 2. Results and Discussion

### 2.1. Generation of Lead Chimeric Peptides

The biology and mechanism of action of the influenza hemagglutinin peptide HA2 is well-known and has been described in detail [12]. Our approach was based on an initial study of the lytic properties of this peptide and some related literature analogs [12]. We also investigated a number of literature CPPs [13]. We initially hypothesized that fusion of a CPP sequence with the membrane active HA2 peptide sequence could provide a chimeric peptide with cellular uptake and endosomal escape properties combined in the same peptide. Simultaneous to our investigations, several other authors reported similar chimeric peptides that were shown to have lytic properties and effect intracellular delivery of cargos [14,15,16,17,18,19]. In addition, fusogenic peptides from this class have been reported to enhance endosomal escape [20]; whether this activity is mediated by direct endosomolysis or not remains uncertain but seems likely. In most cases these investigations focused on the biology of the cellular delivery processes associated with these peptides, but little or no medicinal chemistry was done to specifically investigate the structure –activity relationships required to drive and optimize this activity. We chose a different approach, using a systematic, synthetic analog-driven program to enhance the lytic properties of these peptides to understand and increase their efficiency as possible endosomal escape agents. Since our internal investigations had confirmed that endosomal escape was a highly inefficient process and a key barrier to intracellular delivery, we surmised that we could make the largest direct impact on delivery by enhancing this process.

Initially, we investigated the lytic activity of several CPPs, several influenza hemagglutinin derivatives, and their chimeric fusion peptides (Table 1). Peptides were synthesized using standard FMOC—solid phase synthesis protocols and were obtained as N-terminal amines/C-terminal amides unless otherwise noted (see Methods and Materials for full details). All peptides included a free cysteine reside in the peptide sequences to allow for possible direct conjugation to linkers and potential cargo molecules. To prevent dimerization of the cysteinylated peptides, small amounts of acetic acid and DTT were added to the stock solutions and assay buffer to ensure that the peptides were tested as monomers; the presence of monomers was randomly confirmed by LC/MS analysis of the assay solutions. All peptides were shown to be greater than 95% in purity by HPLC analysis and demonstrated the correct masses by HRMS analysis. Peptides were tested for lytic activity in a standard red blood cell hemolysis assay at pH 7.4 and 5.5 using a modified version of published protocols (see Methods and Materials). This assay is widely considered to be one of the better assays for evaluation of the lytic properties of synthetic peptides. All the peptides tested were freely soluble at up to 10 μM concentrations in the stock solutions and assay buffers, and only the most lipophilic peptides exhibited limited solubility at higher (>10 μM) concentrations. Consequently, solubility does not appear to have played a role in the observed behavior of most of the peptides.

**Figure 1:**
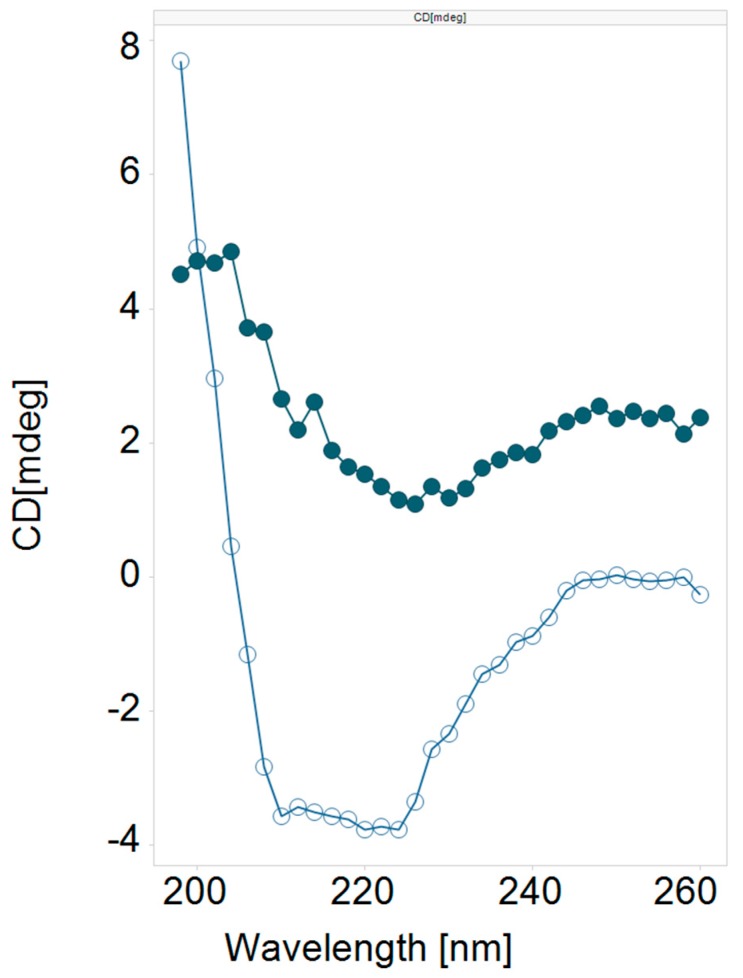
CD Spectrum of Peptide **6**. Filled circles = pH 7.4; open circles = pH 5.5.

We found that the literature cell penetrating cargo delivery peptides **1** (Tat) [21,22] and **2** (Penetratin) [22] were devoid of lytic activity at both pH 7.2 and 5.5. We also found that the parent influenza hemagglutinin peptide **3** (HA2) had weak but detectable lytic activity at either pH, while the HA2 analog **4** (INF7) [23] showed only a trace of lytic activity at pH 5.5. Given that these types of peptides have been shown in the literature to safely effect cargo delivery to the cytosol, we were encouraged by the potential of optimizing this inherent but weak lytic activity. When peptides **1** and **3** were fused together to create the chimeric peptide **5** (HA2-Tat) [14,15,18,19] a large increase in lytic potency was observed at both pH, with about 3-fold selectivity for the lower pH. This was a quite remarkable finding, indicating that something about the combination of the two peptide sequences was conferring potent lytic activity that was not present in either of the two individual peptides. We prepared several analogs (Table 1, **6**–**9**) of the lead chimeric peptide by combining several known HA2 analogs with Tat and found varying degrees of potent lytic activity with each of these peptides. We theorized that the selectivity of the peptide and its pH responsiveness was governed directly by the pKa of the protonatable groups in the peptide, with the mixture of protonatable sidechains in peptide **6** providing the best overall profile. Peptide **6** (INF7-Tat) [24,25] was the most potent of these analogs at pH 5.5, while also maintaining the 3-fold pH selectivity of the lead peptide, and this peptide also demonstrated far superior physical properties and solubility behavior versus the others, and as such became our lead peptide for further optimization. We also prepared the chimeric fusion peptides of HA2 and INF7 with Penetratin (Table 1, **10**–**11**), but surprisingly both were devoid of lytic activity, again confirming the uniqueness of the combination of Tat and the HA2—like peptides. A preliminary CD (circular dichroism) analysis (Figure 1) of **6** showed that the peptide had a random coil conformation at pH 7.4, however at pH 5.5 the peptide was strongly alpha-helical, suggesting that the pH switching mechanism that we hoped to build into the peptides and optimize was indeed present [26,27].

### 2.2. SAR and Optimization of Lytic Activity for Lead Peptide 6

**Figure 2:**
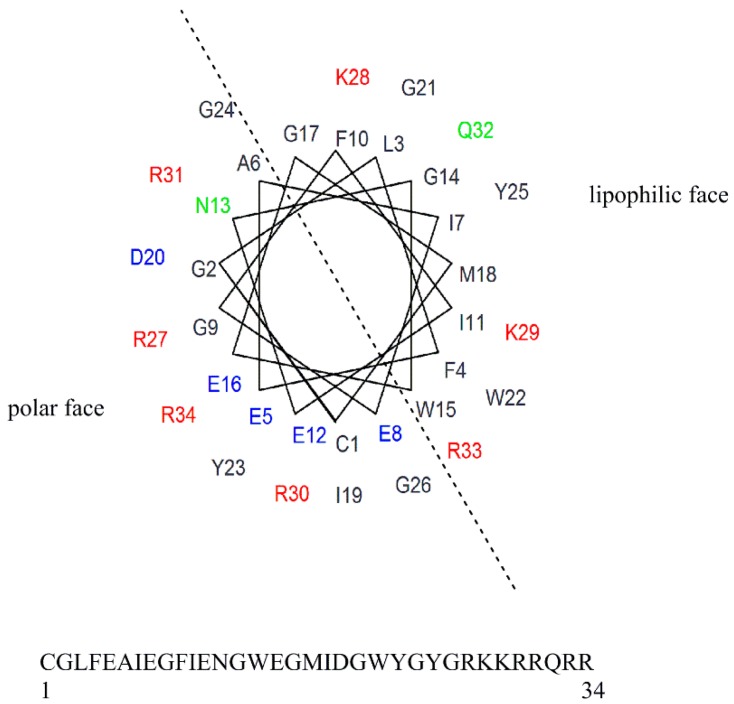
Helical wheel projection of HA-2 Tat.

To fully examine the SAR surrounding the lead peptide **6**, as well as optimize the peptide for lytic activity and selectivity, we began a position-by-position amino acid walkthrough across the entire peptide sequence. Each amino acid in the INF7 portion of the sequence was varied individually, and selective changes were made to the Tat portion of the peptide as well. Positions were varied by charge, polarity, and lipophilicity based on their position on either the polar face or the lipophilic face of a helical wheel diagram of the lead peptide **6** (Figure 2). Table 2 summarizes the RBC lysis data for the position-by- position walkthrough of the INF7 portion of the chimeric peptide. The N-terminal residue (cysteine in most cases) was designated as position 1, moving from N-terminus to C-terminus across the peptides. The use of an N-terminal cysteine in the peptides was not in general detrimental for lytic activity. This is quite interesting in the context of previous publications that have suggested that the N-terminal residues of HA2-like peptides are critical for membrane insertion, with a glycine or other similar residues highly conserved and often a requirement for fusion activity [28,29]. In several cases, the cysteine was moved to other positions in the peptide sequence, and this had a minimal effect on lytic potency.

A drastic effect on lytic potency is observed when the glycine at position 2 is modified. Substitution with charged residues **12a**–**b**, lipophilic residues **12d**–**e**, or a polar residue **12g** results in peptides with equal or improved lytic potency and in several cases much improved pH selectivity. In addition, the insertion of a larger “spacer” residue at this position such as a β–alanine (**12f**) provides a substantial increase in lytic potency at lower pH, while also providing much better pH selectivity. Even a large spacer like three glycines (**12c**) does not affect the lytic potency at pH 5.5; however, it does appear to affect the pH selectivity somewhat. As previously described above, the promiscuousness of the activity observed at this position contrasts with previous literature reports describing the N-terminal residues of HA2–like peptides [28,29]. This again points to the uniqueness of these peptides, and strongly suggests a highly disparate SAR for fusion activity and lytic activity, which is solidified by further data generated in our study.

SAR at position 3 (**13a**–**e**) clearly points to the requirement for a lipophilic residue at this position. This is not surprising given the position of this residue on the lipophilic face of the helical wheel projection of the peptide. A similar analysis can be made for position 4 (compounds **14a**–**f**), which also lies on the lipophilic face of the peptide. An interesting exception at this position is the glutamic acid analog **14d** which does appear to be tolerated in this position and appears to provide an improvement in pH selectivity.

Position 5 (compounds **15a**–**e**) appears tolerant to most polar and charged residues. Again, this is not surprising, given its location on the polar face of the helix, clustered among several charged residues.

**Figure 3:**
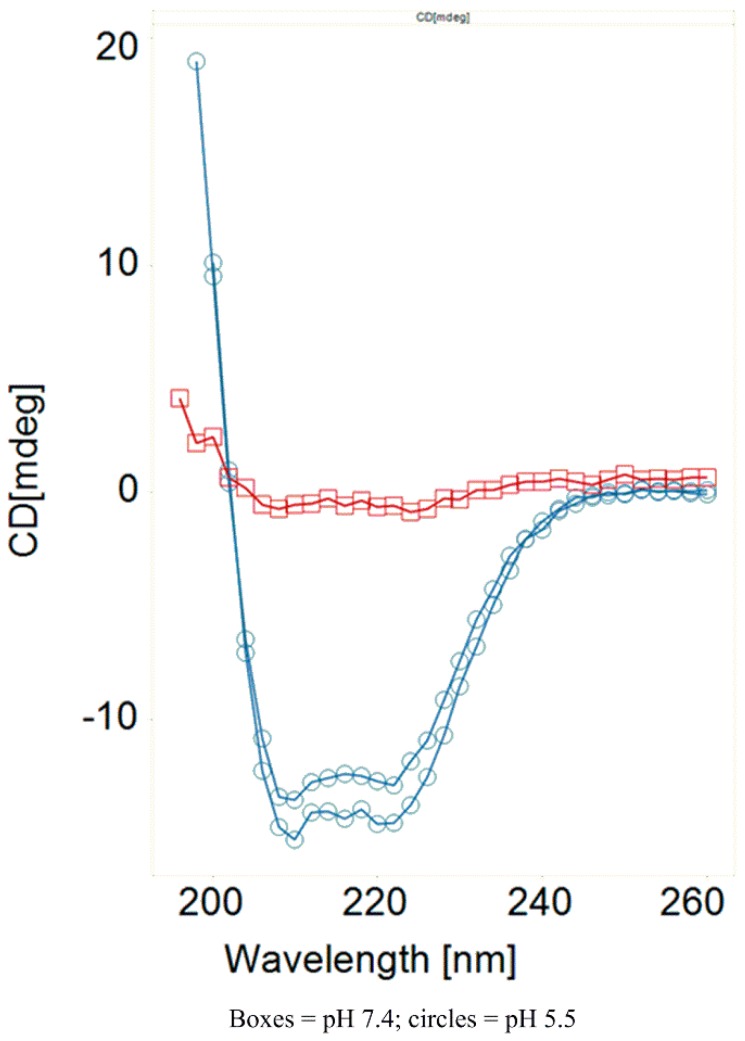
CD spectrum at pH 7 and 5.5 for peptide **16c**.

The replacement of a glutamic acid at this position with a basic lysine residue (**15e**) confers increased potency, but compromises selectivity. This is a trend observed at multiple positions bearing glutamic acids across the peptide. Position 6 lies on the interface between the polar and lipophilic faces of the peptide, and as such analogs at this position demonstrate tolerability for both charged (compounds **16c**–**d**) and lipophilic (compound **16b**) residues. The glutamic acid analog at this position (**16c**) is especially noteworthy, providing a 5-fold enhancement of lytic potency at pH 5.5, and excellent pH selectivity. Figure 3 shows a comparison of the CD spectrum of **16c** at pH 7 and 5.5, providing clear conformational support for the high degree of selectivity observed with this peptide. This peptide is one of the best performing single amino acid modifications identified in this study and highlights the critical importance of this position for lytic activity. Interestingly, the asparagine analog at this position is not well tolerated and loses all lytic potency. This is an interesting observation and is somewhat difficult to rationalize given the other SAR at this position.

Position 7, occupied in the lead peptide by an isoleucine residue and lying in the center of the lipophilic face of the peptide helix would be expected to be highly intolerant of polar or charged residues, and the SAR directly confirms this. The leucine analog at this position **17c** provides a peptide with enhanced potency at lower pH, as well as improved pH selectivity. The charged, basic lysine analog **17a** loses all lytic potency at both pH. Once again, as seen with the similar position 4, a glutamic acid (compound **17b**) is somewhat tolerated in this position along the lipophilic face of the helix.

In a similar manner to position 6, position 8 lies along the interface between the polar and lipid faces of the peptide helix. As such, this position appears to be one of the most tolerant positions in the entire peptide, able to be replaced by lipophilic/aromatic (**18a**), charged (**18b**–**e**), or small/no sidechain (**18f, 5**) containing amino acid residues. Only the histidine analog (**18c**) at this position produced a reduction in lytic potency at pH 5.5. In terms of pH selectivity, there was no clear trend, but all the analogs were either balanced in terms of pH selectivity or showed improved selectivity versus the lead peptide.

Position 9 of the peptide lies along the polar face of the peptide helix and as such would be expected to tolerate polar and charged residues **19a**–**c**, and this is confirmed by our data. This position surprisingly is also tolerant of a lipophilic residue (**19d**), in a similar manner to the glycine 2 residue that is located close by on the peptide helix. Also, in a similar manner to glycine 2, a glutamic acid residue (compound **19c**) is well tolerated at this position and provides a 3-fold increase in lytic potency at pH 5.5 as well as an increase in pH selectivity. The fact that these two glycine residues are close to each other on the peptide helix and share some common SAR features may indicate that this region of the peptide is acting as a unit to govern overall peptide behavior.

Position 10 of the peptide lies on the lipophilic face of the peptide helix and our SAR analysis at this position indicates a strong preference for non-polar/non-charged residues. Glycine (**20a**), alanine (**20b**), and leucine (**20c**) each provide increasing lytic potency at pH 5.5 as the size/lipophilicity increases of the sidechain. Interestingly, a similar trend is seen at pH 7.4, with lytic activity increasing with sidechain size/lipophilicity. Unlike several other residues in this region, neither acidic (**20e**) nor basic (**20f**) amino acid sidechains are tolerated at this position.

SAR at position 11 of the lead peptide also appears to be largely governed by its position on the lipophilic face of the peptide helix. The glycine substitution (**21a**) at this position provides a peptide devoid of lytic activity, however gradually increasing the size of the substituent here from a methyl (alanine; **21b**) to a larger carbon chain (leucine; **21c**), to finally a large aromatic substituent (tryptophan; **21d**) results in concomitant increases in lytic potency at pH 5.5. In each case, selectivity is maintained or increased, indicating that this position may be an important handle for introducing increased pH selectivity into the peptide. Replacement with a polar sidechain (asparagine; **21e**) or a charged sidechain (lysine; **21f**) results in complete loss of lytic activity at both pH.

Position 12 of the parent peptide is located on the polar face of the helix, in an area that appears to be a cluster of glutamic acid residues. Interestingly, replacement of this glutamic acid residue with lipophilic residues such as a leucine (**22a**) or a tryptophan (**22b**) produces peptides with equal or improved lytic activity at lower pH; however, the peptides become equipotent at physiological pH as well. A basic lysine residue (**22c**) or an unsubstituted glycine residue (**22d**) at this position produced peptides that retain some lytic activity at pH 5.5, although the activity is diminished from the parent peptide.

Positions 13 and 14 define a region of the INF7 portion of the peptide that has been described by previous authors as a “hinge region” in HA2 peptides [30,31,32], providing flexibility between separate N-terminal and C-terminal helical regions and allowing the peptide to form a “boomerang” or U–shaped conformation that is theorized to aid the peptide in penetrating biological membranes for fusion activity [30,31,32]. We incorporated several amino acids at each of these positions that were designed to test this hypothesis. Theoretically, a proline at position 13 might serve as an excellent mimic of such a hinge; however, we found that substitution of proline (**23a**) at this position produced a complete loss of lytic potency. Substitution with glycine at this position (**23b**), which may also be able to support a hinge mechanism, was better tolerated and provided a profile equal to the parent peptide. Increasing the size of the amino acid sidechain at this position from leucine (**23c**) to tryptophan (**23d**) provided peptides with equal or better lytic potency at pH 5.5 versus parent. Charged residues such as lysine (**23e**) or glutamic acid (**23f**) also provided peptides with equal or better lytic potency at pH 5.5 with good selectivity versus physiological pH. Position 14 showed a somewhat different profile, with highly charged residues such as glutamic acid (**24a**) or lysine (**24b**) not well tolerated, but less charged/polar residues such as asparagine (**24c**) or histidine (**24d**) showing moderate lytic potency. In contrast to position 13, proline (**24e**) was well tolerated, showing a slight loss in potency at lower pH with only slight pH selectivity observed. Interestingly, replacement of the glycine with an alanine (**24f**) led to a peptide with a potent, balanced lytic profile, while replacement with the lipophilic/aromatic tryptophan (**24g**) produced a peptide with lytic potency similar to parent at acidic pH, with improved pH selectivity.

The tryptophan residue at position 15 lies on the lipophilic face of the peptide in a helical projection, very close to the interface/transition between the lipid and polar faces of the peptide. Interestingly, replacement of the tryptophan with a glutamic acid (**25a**) produces a peptide that exhibits an inverted pH profile. This peptide was one of the most lytically potent peptides we observed at physiological pH. Changing the charge at this position from negative to positive as in the lysine replacement (**25b**) provided a peptide that was inactive at both pH. This position appears to be quite important for pH selectivity as demonstrated by these two oppositely charged amino acid analogs. Simply replacing the tryptophan with a lipophilic but non-aromatic leucine residue (**25c**) provided an approximately two-fold increase in lytic potency at acidic pH while also improving the pH selectivity.

Position 16 appears to be one of the more tolerant positions along the peptide backbone and was largely tolerant of all amino acid changes that were made. Simple modification of the glutamic acid to an aspartic acid provided a peptide with several fold enhanced potency at pH 5.5 (**26a**). A positively charged residue at this position (**26b**–**d**) also maintained some level of potency and selectivity, with the lysine sidechain at this position (**26c**) providing one of the overall best profiles of all peptides in the study. A non-charged, polar residue such as asparagine maintained good potency at pH 5.5 with enhanced pH selectivity (**26e**)**.** Small alkyl sidechains (compounds **26f**–**g**) at this position maintained some level of lytic potency and pH selectivity, while even a glycine (**26h**) maintained some level of potency at acidic pH with good pH selectivity. Several the modifications at this position demonstrated complete pH selectivity, confirming this residue as another key position for maintaining the desired pH balance.

Modification of the glycine residue at position 17 provided several interesting novel peptides. Substitution with an alanine residue at this position (**27a**) provided a peptide with enhanced potency at pH 5.5, but a blunted maximal lytic effect. Addition of a charged sidechain at this position, whether negative (**27b**) or positive (**27c**) provided peptides that maintained lytic potency at pH 5.5 but showed somewhat enhanced pH selectivity. Interestingly, the asparagine analog (**27d**) demonstrated a very nice overall profile, with enhanced potency at lower pH and improved pH selectivity.

The methionine residue at position 18 was of particular interest to us, since we had seen some propensity for oxidation to occur at this position on storage of the peptide in solution. Consequently, we were eager to explore alternative amino acid substitutions at this position. We prepared samples of both potential oxidation products at this position, and both the sulfoxide (**28a**) and sulfone (**28b**) were completely devoid of any lytic activity at either pH. Interestingly, if one looks at the helical projection of the peptide, this residue lies near the center of the lipophilic face of the peptide, and consequently polar substitutions would be likely to be detrimental to stability of the helix and in turn lytic activity. Confirming this hypothesis, replacement of this residue with polar, uncharged sidechains (**26c**–**f**) resulted in peptides that were lytically inactive at both pH. Intuitively, one would also expect that lipophilic sidechain modifications at this position would much better tolerated, and that does appear to be the case. Substitution with either a leucine (**28g**) of a norleucine (**28h**) provided peptides with enhanced lytic potency at pH 5.5 and similar selectivity to parent. Aromatic sidechain-containing residues such as tyrosine (**28i**) and phenylalanine (**28j**) were also well tolerated, providing equal or better lytic potency at pH 5.5 with somewhat enhanced pH selectivity. As expected, complete deletion of the residue (**28k**) provided a peptide with only very weak lytic activity, presumably due to the disruption of the overall amino acid sidechain arrangement of the helical form of the peptide.

Replacement of the isoleucine at position 19 provided a somewhat interesting SAR. Replacement of this highly lipophilic, branched residue with a very similar leucine residue (**29a**) surprisingly resulted in a loss in lytic potency. Shrinking the sidechain even more to an alanine (**29b**) resulted in a complete loss of lytic activity. However, completely removing the sidechain and substituting with a glycine residue (**29c**) restored the lytic activity to near the level of parent, with enhanced pH selectivity also observed. Substitution with an uncharged polar residue (**29d**) provided a peptide with improved lytic potency at pH 5.5 and improved pH selectivity. Substitution with a positively charged lysine residue (**29e**) provided a peptide with an almost identical profile; however, substitution with a negatively charged residue (**29f**) resulted in a complete loss of lytic activity. It is difficult to rationalize the observations made by changing the sidechain residues at this position, other than to speculate that if one looks at a helical projection of the peptide, this residue lies close to the interface between the lipophilic and polar faces of the amphiphilic helix, and this may in part help to explain the somewhat contrary SAR observed for this position.

Position 20 appears to be one of the least tolerant positions in the peptide sequence. Modification from glutamic to aspartic acid at this position (**30a**) resulted in a loss of lytic potency, and substitution with small (**30b**) or large (**30c-d**) lipophilic sidechains completely obliterated lytic activity.

Contrary to the observation for position 20, position 21 appeared highly tolerant of substitution and proved to be an excellent position for enhancing lytic potency at pH 5.5 as well as optimizing pH selectivity. Substitution with alanine at this position (**31a**) provided a peptide with equal lytic potency to parent at pH 5.5 but enhanced pH selectivity. Adding a larger lipophilic sidechain such as leucine (**31b**) provided enhanced lytic potency at low pH while maintaining a similar selectivity ratio to parent. Interestingly, substitution of isoleucine at this position (**31c**) provided similar lytic potency to parent at pH 5.5, but greatly enhanced pH selectivity. Substitution with the larger lipophilic/aromatic sidechain of tryptophan (**31d**) provided a peptide with enhanced lytic potency at pH 5.5 and improved pH selectivity; this individual peptide had one of the more favorable overall profiles of all the peptides tested. Substitution with a polar, uncharged asparagine residue (**31e**) resulted in a reduction of lytic potency and selectivity, however charged polar residues such as lysine (**31f**) or glutamic acid (**31g**) were tolerated with equal lytic potency at pH 5.5 and improved pH selectivity.

Positions 22–24 compose the “WYG addition” to the C-terminus of the INF7 portion of the chimeric peptide. Addition of the WYG residues that are present at the C-terminus of the native HA2 fusogenic peptide to numerous similar peptides has been reported in the literature [33] to provide increased membrane affinity and fusogenic potency, so we sought to incorporate this into our sequences from the beginning of our investigations. Indeed, two of the first peptide sequences that we prepared were the parent with and without the WYG addition. Interestingly, removal of the WYG insert from the parent chimeric peptide (**32a**) resulted in a complete loss in lytic potency, suggesting the critical importance of this small addition to the peptide. The uniqueness and specificity of this small insert into the parent chimeric sequence was confirmed by simply substituting a GGG spacer for this sequence. This peptide (**32b**) was also lytically inactive. It is difficult to infer why these three amino acid inserts are so critical to the activity of these peptides. If one compares the helical distribution of the peptides with and without these three amino acids, both peptides are remarkably similar in their amphiphilicity and amino acid distribution. The peptide with the WYG insert does add an additional tryptophan to the lipophilic face of the peptide in a region that is quite close to another tryptophan residue at position 15, and there has been some experimental evidence published [33] suggesting that this residue is a key residue for anchoring the HA-2 family peptides in the membrane. So, it is possible that the additional tryptophan may aid in this membrane anchoring process, however this remains highly speculative and there is no definitive experimental evidence to support this hypothesis.

The individual residues at positions 22–24 were also varied in an independent manner as with the rest of the peptide. Position 22 proved to be somewhat intolerant to various changes. Substitution with a smaller, non-aromatic but lipophilic leucine sidechain (**33a**) resulted in a peptide with an inverted pH profile, and further reduction of the size of the sidechain to a methyl (**33b**) resulted in lower in lytic potency at both pH. Completely removing the side chain (**33c**) by substitution of a glycine at this position eliminated lytic activity at both pH. Substitution with a polar, non-charged asparagine residue (**33d**) provided a peptide with reduced potency at both pH, but a favorable overall selectivity profile. Substitution with either a positively charged lysine residue (**33e**) of a negatively charged glutamic acid residue (**33f**) provided peptides with reduced lytic potency. In general, the tryptophan at this position does indeed appear to be very important for maintaining lytic activity.

Variation at position 23 also provided some interesting and somewhat surprising results. Shrinking the sidechain to a methyl (**34a**) resulted in a complete loss of lytic potency. Replacement of the aryl sidechain with a heteroaryl imidazole (**34b**) provided a peptide with reduced lytic potencies at both pH. Interestingly, substitution with an asparagine residue at this position (**34c**) resulted in a ten-fold increase in lytic potency at pH 5.5, and a corresponding three-fold increase at physiological pH. A glutamic acid substitution lowered lytic activity at both pH but provided a peptide with a favorable overall pH profile.

Several single amino acid variants at position 24 were also examined. Modification to an alanine at this position (**35a**) resulted in a peptide with similar lytic activity at pH 5.5 to parent but improved pH selectivity. Modification to a large lipophilic aromatic residue (**35b**) at this position provided a peptide with apparently enhanced lytic activity at both pH; however, the peptide did not provide maximum activity at lower pH. A similar profile was seen for substitution with a glutamic acid at this position (**35c**), with the peptide providing only 60% maximum lytic activity at lower pH.

Beyond the systematic single amino variants at each position of the INF7 portion of the chimeric peptide, we also investigated several other important peptide analogs. The results for these subsequent peptide analogs are summarized in Table 3. One series of analogs that we wanted to consider was the possibility of inserting a spacer between the INF7 and Tat portions of the chimeric peptide. Moving the position of the cysteine residue from the N-terminus to the center of the peptide (**36a**) provided a peptide with a balanced lytic profile. Inserting a GGG spacer between the two component peptides (**36b**) provided a peptide with two-fold enhanced lytic potency at pH 5.5, and improved pH selectivity versus physiological pH. Given this interesting result, we attempted to make the spacer between the two individual peptide chimeras larger by inserting amino/acid terminated peg spacers. Quite remarkably, good lytic potency and pH selectivity were maintained for (peg)3 (**36c**) and (peg)6 (**36d**) spacers, and even the (peg)11 (**36e**) spacer showed a reasonable overall profile. Pushing this to a larger (peg)27 (**36f**) spacer eliminated lytic activity.

While we did not individually vary the residues in the Tat portion of the chimeric peptide, we did make several interesting changes to this region of the peptide that resulted in favorable contributions to the overall SAR. To favorably alter the overall pH selectivity profile of the parent peptide, we focused on examining multiple changes to the basic, protonatable residues present in the Tat potion of the chimeric peptide. Replacement of all five of the arginine residues with lysines (**37a**) provided a peptide with reduced lytic potency at both pH. Similarly, replacement of all the arginines with histidines (**37b**) provided a peptide with slightly improved lytic potency at lower pH, but improved pH selectivity. Adding an additional arginine to the C-terminus of the parent (**37c**) sequence provided a peptide with a largely identical lytic profile. Interestingly, removal of one of the arginines from the C-terminus (**37d**) provided an increase in lytic potency at lower pH, along with an improvement in pH selectivity. Given this result, we systematically removed C-terminal residues one by one until we began to see a detrimental effect on lytic activity. Removal of two arginines (**37e**), or even of the last three residues (two arginines and a glutamine; **37f**) provided peptides that maintained good profiles. It was only when the four consecutive C-terminal residues were removed (**37g**) that a large drop off in lytic potency was observed. Replacing four of the five Tat arginine residues with histidine but maintaining the C-terminal arginine (**37h**) provided a peptide with one of the most favorable overall pH profiles that we saw in the entire study, exhibiting a three –fold increase in lytic potency at pH 5.5 and enhanced pH selectivity. This peptide was soluble enough that we were able to push the concentration up to as high as 100 μM at physiological pH and saw no lytic activity at this concentration. Once again as stated earlier, the pH responsiveness of the overall chimeric peptide can clearly be changed by altering the pKa and number of protonatable groups present in the peptide. However, being able to control this in a systematic, predictable manner proved to be quite challenging, and once again our efforts were driven primarily by intuition and empirically observed data.

We also examined several other overall variants of the parent chimeric peptide sequence. Simply moving the cysteine residue from the N-terminus of the peptide to the C-terminus of the peptide (**38a**) provided a peptide with a slightly improved overall pH profile over parent. Simple retro-analogs (**38b**,**c**) with the cysteine at either the C-or N-termini showed similar profiles to parent and **38a**. The all (D) version of the peptide (**38f**) once again showed a similar profile, while the retro-inverso peptides (**38d**,**e**) showed lower lytic potency at pH 5.5. Interestingly, the similar activity observed for the all (D) peptide strongly suggests that the lytic activity seen with these peptides is likely a function of the alpha-helical, amphiphilic nature of these peptides, is independent of the overall chirality of the peptide analogs and suggests that these effects are independent of direct interactions with any specific binding sites. This may also be important in terms of metabolic stability; the harsh environment of the late endosome is likely a very unfavorable environment for metabolically unstable peptides, and the all (D) versions likely offer substantial advantages in these regards.

Given that the membrane disruptive/lytic activity observed with these peptides strongly appeared to be driven by physiochemical properties such as helicity and amphiphilicity, we wanted to prepare some stapled analogs of these peptides to see if the incorporation of a hydrocarbon staple across the lipophilic face of these peptides would make the transition from a more random conformation to an alpha helical conformation more favorable (Table 4) and thus improve lytic potency and/or selectivity. By simply looking at the helical wheel representation of the parent peptide (Figure 2), several potential target stapled peptides were identified. We chose to initially try stapling between the following residues using *i,i+7* hydrocarbon staples: L3 and F10, F4 and Ill, and Ill and M18. We consistently used the same amino acid side-chain lengths for each analog (5 carbon terminal olefin for the more N-terminal residue, and 8 carbon terminal olefin residues for the more C-terminal residue of the staple) as suggested by the literature [34,35,36] and followed established literature guidelines [34,35,36] for stereochemistry at each residue. Hydrocarbon stapled peptides were synthesized using standard Fmoc-solid phase peptide chemistry, with the metathesis reaction performed on resin using Grubbs Gen. II catalyst [34,35,36], followed by deprotection and cleavage. All three hydrocarbon stapled analogs (**39a**–**c**) showed impressive lytic activity at pH 5.5 that was equal to or better than parent with good pH selectivity. However, compound **39b**, which had the hydrocarbon staple spanning from residue 4 to residue 11 showed one of the best overall profiles of all the peptides tested in this study. This result further strengthens our hypothesis regarding the critical importance of the amphiphilic, alpha-helical character of the peptides for achieving potent lytic activity. Unfortunately, these stapled derivatives proved quite difficult to work with due to inherent issues with solubility and aggregation, as well as poor synthetic yields coupled with difficult purifications. Nonetheless, the constraint of peptides in this class using hydrocarbon staples appears to be a promising strategy for enhancing lytic potency and pH selectivity, and further studies to take advantage of this strategy while also enhancing the physical properties of the peptides are in progress and will be reported in more depth in future publications.

One other modification that we considered was the addition of lipid chains to the termini of the parent peptide (Table 4). Theoretically, the addition of a lipid to the peptide could aid in interaction with and insertion into lipid membranes. To add a lipid at the C-terminus, we added a lysine residue to the C-terminus of the parent peptide and acylated the sidechain amine of the lysine with stearic acid to provide peptide **39d** (Table 4). This peptide showed an interesting, highly lytic profile that was potent and balanced at both pH. Acylation of the N-terminus of the peptide directly with stearic acid and shifting the cysteine conjugation point to the C-terminus provided peptide **39e**, which showed two-fold better lytic potency at pH 5.5 and improved pH selectivity. Unfortunately, once again the extremely poor physical properties of the peptides limited the effort in this area.

Of course, after these extensive SAR studies, the clear question becomes how much of this SAR can be combined in an additive or synergistic manner to produce more lytically potent and pH selective peptides. There are literally thousands of possible permutations and combinations of the observed SAR, and we will highlight just a few of the most interesting combination analogs here. In general, we tried to combine some of the most interesting single amino acid changes to look for additivity or synergy.

From the results in Table 3, there are several interesting trends. In general, potency at pH 5.5 is in almost all cases enhanced, while pH selectivity in almost all cases is also better than parent. So, the overall trend is toward peptides with enhanced profiles. However, the specifics of the combined modifications are quite varied, and peptides with several very different profiles are observed by combining various amino acid changes. While the general trend was toward better peptides, the specific combinations were not always additive or even mutually tolerated. Interesting peptides were found by combining as few as two amino acid changes, or as many as nine amino acid changes (Table 5).

One of the most interesting peptides in the entire study (compound **53**) was obtained by combining optimal modifications at positions 15, 16, 18, and 21; this peptide is among the most lytically potent peptides tested at pH 5.5, while demonstrating excellent pH selectivity. In terms of raw potency at pH 5.5, several peptides with five amino acid changes from parent were observed to possess sub-100 nM potency (compounds **56** and **58**), while maintaining respectable levels of pH selectivity. This represents a greater than 20-fold enhancement of lytic potency at pH 5.5 by simple substitution of five key amino acid residues. Even peptides with as many as nine (**62**) amino acid changes maintained respectable overall profiles that were better than parent.

We were intrigued by the sequence of peptide **61** (Table 5), which maintains good lytic potency at pH 5.5 and good pH selectivity despite having many anionic residues in the sequence. While the parent HA-2 type and other fusogenic peptides are anionic in nature, and there are known cell penetrating [37] or membrane active/antimicrobial peptides [38,39] that are largely anionic, most peptides in this space are typically highly cationic peptides. To further probe the relationship of charge to lytic activity and pH selectivity, we prepared a group of analogs based on the sequence of **61**, in which we made additional amino acid substitutions using the data gathered from our amino acid walk-through as a guide (Table 5). Combining the INF7 portion of **61** with the histidine-modified Tat from **37h** provided peptide **62**, which maintained lytic potency at pH 5.5 while providing full pH selectivity. This peptide is also noteworthy in that it has 14 amino acid positional changes from the original lead structure and maintains good lytic potency and pH selectivity. Substituting additional negatively charged residues into the sequences of **61** and **62** at position 16 provided peptides **63** and **64**. Although both peptides lost some lytic potency, they still retain a reasonable level of lytic activity. This is quite interesting in that peptides **63**–**64** both possess an overall net charge of zero, having 7 positively and seven negatively charged residues in their sequences. These peptides represent the first peptides that we have identified in this series that maintain lytic activity without having an overall positive charge. Similar analogs **64**–**67** which have slightly modified sequences maintain the overall net zero charge and exhibit quite similar potency and selectivity to **63** and **64**. Investigating this further to insert an additional negative charge provides peptide **68**, which continues the trend towards reduced lytic potency but remarkably still retains some level of lytic activity and selectivity even with an overall negative charge on the peptide. Again, combining the features of **68** with the histidine-modified Tat from **37h** provided peptide **69**, which showed an additional potency loss. However, simply removing one of the negatively charged residues at position **16** improved the lytic potency of the peptide at pH 5.5 back into a reasonable range while maintaining good pH selectivity. While the lytic activity of peptides **62**–**70** is diminished versus the most potent analogs observed in this study, the SAR clearly suggests that negatively charged residues can act in a similar manner to positively charged residues to help drive membrane disruption. This finding once again suggests that overall charge distribution and amphiphilicity likely play a greater role than the actual charge itself in the observed lytic activity. It is also noteworthy that peptides such as **61**–**70** may constitute novel and unique lytic/membrane active peptide space, and as such it may be possible to use these sequences as next generation leads for further reengineering or optimization.

**Figure 4:**
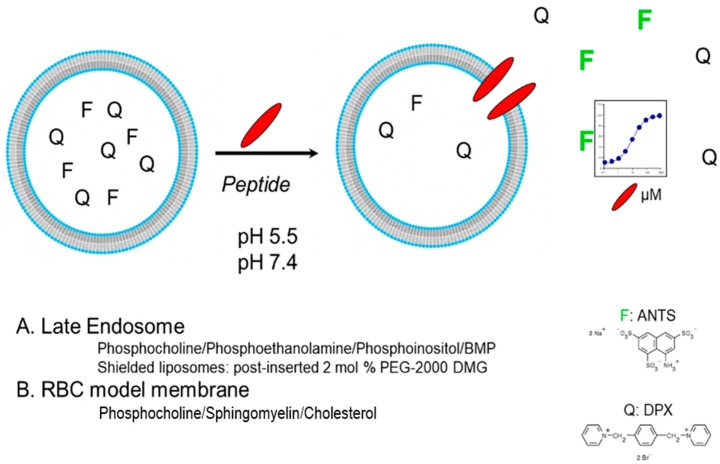
Liposomal leakage model.

**Figure 5:**
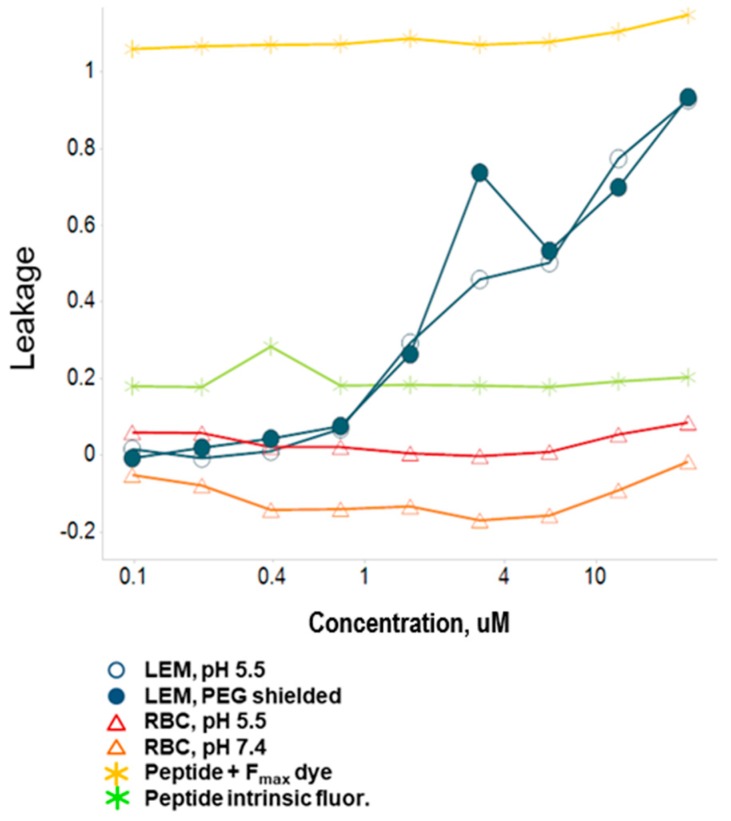
Liposomal leakage data for peptide **38f**.

To elucidate more information regarding the mechanistic aspects of the interaction of these peptides with membranes, we investigated the effects of peptide **38f** on extruded multilamellar vesicles in a liposomal leakage model where the liposomal membranes were designed to mimic either RBC or late endosomal membranes. Late endosomal model membranes were prepared using the combination of phosphocholine/phosphoethanolamine/phosphoinositol/BMP, while RBC-mimicking membranes were prepared using phosphocholine/sphingomyelin/cholesterol. We also prepared “shielded” (PEG coated; post-inserted 2 mol% PEG-2000 DMG) late endosomal liposomes designed to prevent fusion events between vesicles. Liposomes were constructed to contain an ANTS-dye and a DPX -quencher (Figure 4), and the liposomes were treated with a solution of the test peptide at pH 5.5 (late endosomal pH) and pH 7.4 (physiological). Upon lysis of the liposomal membrane, the ANTS dye is freed and on separation from the DPX quencher produces a fluorescent signal which can be quantified to provide the level of leakage observed. Figure 5 summarizes the results for peptide **38f.** A clear separation is seen between the leakage activity demonstrated against the late endosomal membrane (LEM) liposomes versus the RBC-mimicking liposomes, with the peptide demonstrating selectivity for the LEM-mimicking liposomes. Leakage from LEM is clearly evident with some observation of fusion/aggregation of these liposomes; coating the liposomes with PEG prevented the fusion and aggregation events but did not prevent leakage. This data suggests that the leakage event is primarily driven by peptide insertion and direct disruption of the endosomal membranes, rather than by “leaky” endosomal fusion events. As stated earlier, we believe that the peptides undergo a pH/protonation-driven conformational change at late endosomal pH, causing them to snap into an alpha -helical conformation, which we believe is the active species that inserts into the membrane leading to disruption and ultimately lysis of the endosomal membranes. Interestingly, we observed an almost “on-off” switch-like behavior for these peptides. The peptides remain inactive until they reach a threshold concentration, as which point leakage begins and rapidly increases. This mass-driven effect is seen often with various classes of membrane active peptides and is suggestive of a carpet-like mechanism of membrane disruption in which the peptides coat the membrane surface and insert into the membrane causing a generalized, detergent-like disruption of the membrane integrity. However, the observed selectivity for late endosome-like membranes is quite interesting. Most cationic, membrane disruptive peptides that behave in a carpet-like mechanism are known to be generalized membrane disruptors with little selectivity. It is likely that in the case of these peptides, the highly amphiphilic, alpha-helical conformation allows for efficient insertion into membranes while the sequence and display of the charged and lipophilic residues is perfect to induce a preference for direct interaction with the more negatively charged lipids of late endosomal membranes.

One might ask if these peptides in general possess cell-penetrating/cargo delivery properties. While this was not the focus of our studies, and we will not detail specific data regarding this, in general we found that these peptides were quite inefficient as classical cell penetrating peptides/cargo delivery peptides. This is not surprising, given that the focus of our SAR studies was to design peptides with maximized membrane disruptive capabilities and was not focused on cargo delivery. Our in—house studies with these peptides and several other classical cell penetrating peptides suggests a huge diversity of properties and behaviors for this entire class of peptides. We have found in general that the cell lines and conditions under which many cell penetrating/cargo delivery peptides are tested largely govern the behavior observed with them and attempts to define the mechanisms of these peptides or to find general rules that could be applied across the board to new peptide design were largely inconclusive. By synthesizing a simple chimeric fusion of two very different membrane active peptides and systematically optimizing for lytic potency and selectivity, we obtained novel peptides with profiles quite disparate from that exhibited by the individual peptides. Our results suggest that that various classes of membrane active peptides likely have quite differing structure-activity relationships, and defining specific, sequence-based rules for novel peptide design would likely prove to be quite difficult. Instead, a more empirical approach employing general consideration of the overall properties and secondary structure of the peptides is likely a better way to optimize membrane interactions. Lipophilicity, charge, amphiphilicity, and helicity appear to be key drivers for these processes. Unfortunately, experimental tools that allow one to make scientifically proven conclusions about specific mechanisms are in general lacking, and results based on flawed models fuel widespread speculation in the field of membrane active peptide/CPP design.

## 3. Materials and Methods

### 3.1. Peptides

Peptides **1**–**10** were synthesized in-house on a CEM Liberty Microwave Peptide Synthesizer (Matthews, NC, USA), using standard Fmoc-chemistry. After cleavage and isolation all crude peptides were purified to >95% purity using reversed phase HPLC (C4, C8, or C18 columns). Stapled peptides **39a**–**c** were synthesized by CPC (Hangzhou, China). All other peptides were custom synthesized externally by Biopeptek (Malvern, PA, USA). Peptides were obtained as N-terminal amines/C-terminal amides unless otherwise noted. All internally and externally synthesized peptides were purified to >95% purity and demonstrated correct masses after HRMS analysis.

### 3.2. RBC Lysis Assay and Analysis

The human red blood cell hemolysis assay was by carried out as described previously [40] with modifications. Briefly, 5 mL aliquots of human blood from healthy individuals were dispensed into 50 mL centrifuge tubes and resuspended in 35 mL of buffer containing 150 mM NaCl and either 20 mM MES adjusted to pH 5.5 or 20 mM HEPES adjusted to pH 7.4. Red blood cells (RBCs) were washed 3 times via centrifugation with buffer and finally resuspended in a total of 50 mL of buffer. For the final assay, 175 μL of buffer solution was dispensed into each well of a clear-bottomed 96-well plate followed by 50 μL of final resuspended RBCs (approximately 2.5 × 107 cells). For transfers of RBCs, wide-bore pipet tips were used to avoid cell damage. Test peptides at the appropriate concentration were first diluted in 25 μL of buffer and then added to the cells. Small amounts of acetic acid and DTT were added to the stock solutions and assay buffer to ensure that the peptides were tested as monomers; the presence of monomers was randomly confirmed by LC/MS analysis of the assay solutions. Serial dilutions of peptides to be tested were prepared in an Easy-Wash (Costar 3368), 96 well plate, in water. Serial dilutions of peptides to be tested were prepared using a multichannel pipet. The standard assay uses a 10 point 2-fold dilution series. Compound dilutions typically are prepared at a 10× (200 μM) concentration in 25 μL final volume. The highest concentration tested was dependent on the material and its respective solubility. Buffer alone and 1% Triton X-100 in PBS were included as negative and positive controls respectively. Dilutions are prepared as either duplicates or single wells depending on the number of samples to be tested and the amount of material available for testing. All steps prior to incubation were carried out with chilled buffers and on ice. Finally, the suspension was mixed with a wide-bore tip, and the plate was covered and incubated at 37 °C for the indicated time. After incubation, the cells were centrifuged for 5 min at 500 rcf, and 150 μL of the supernatant was transferred into a new 96-well clear-bottom plate. Absorbance was measured at 541 nm, and the resulting raw hemolysis figures were normalized to a matching set of RBCs incubated in the presence of 1% Triton X-100 (100% hemolysis control). The known RBC lytic peptide melittin was used as an internal standard in all assays performed. The background absorbance is subtracted from all samples, treating each pH grouping separately. The % hemolysis is calculated for each sample at varied concentrations as a % of the Triton X-100 sample (100% hemolysis), standard curves plotted, and the EC 50 values calculated using ADA. Unless indicated, all peptides were tested a minimum of three determinations at each pH, and the activity is reported as the mean. Standard deviations were determined for the compounds with larger n values, to provide some guidance regarding the variability of the assay.

### 3.3. Circular Dichroism Spectra

CD spectra were collected using a VPR706 spectropolarimiter (Jasco, Easton, MD, USA). The following instrument parameters were used:

Band width1 nmResponse2 secSensitivityHighMeasurement range300–200 nmData pitch1 nmScanning speed50 nm/minAccumulation1 or 3 scans as notedCell Length0.1 cmTemperatureAmbient

Peptides were dissolved in a minimum of TFE and were diluted into the appropriate buffer (pH 7.4 = PBS; pH 5.5 = MES) to give a final concentration of 25 μM. Runs are the average of three scans. Samples were prepared by mixing peptide stock solution and buffer directly in a 0.1 cm × 1 cm, 0.5 mL quartz cuvette. Cuvette was cleaned with an aspirating washer with dd-water and methanol until dry.

## 4. Conclusions

In summary, a systematic analysis of the sequence of the chimeric INF7-Tat peptide has demonstrated a clear SAR for lytic activity and pH selectivity. This SAR appears to be quite unique to the chimeric nature of these peptides and is disparate from previously observed SAR for the individual peptides. The observed lytic potency and selectivity has been shown to be optimizable by manipulating the pKa of the peptide sidechains to refine the point at which the peptides change secondary structure from a random coil to an alpha-helix. Simultaneously, enhancing/maximizing the overall amphiphilic nature of the peptide helix through targeted modifications of the appropriate lipophilic and charged polar residues also appears to be a key contributor to lytic potency. Several modified peptides were shown to have greatly enhanced lytic potency at late endosomal pH of 5.5 while also possessing much enhanced selectivity versus lysis at physiological pH. This study represents one of the first reports of a highly systematic, position-by-position SAR-based approach to the design and optimization of novel membrane active peptides. Our study clearly demonstrates that direct manipulation of the charge, lipophilicity, and overall amphiphilicity of helical, membrane active peptides can be used in a systematic manner to optimize their potency and pH selectivity. This general set of criteria can be useful to systematically optimize other classes of membrane active peptides such as antimicrobial peptides, CPPs, and various other endosomal escape peptides. Our data suggest that using a systematic, position-by-position optimization strategy for membrane active peptides may lead to significant improvements in activity, while also helping to develop novel chemical space that otherwise would remain largely unexplored. Based on the encouraging results of this preliminary effort, we are continuing to develop novel peptides that may be useful for delivery by enhancing endosomal escape. We are extending these design principles to the investigation of next generation peptides with novel charge distributions and enhanced endosomal escape properties that are membrane active but not membrane disruptive, and as such are less likely to cause safety issues than these highly lytic first-generation analogs. We will report on our continued efforts in due course.

## Figures and Tables

**Table 1:** Lead Chimeric Peptides.

Number	Sequence	RBC Lysis IC_50_, pH 5.5 (μM)	RBC Lysis IC_50_, pH 7.4 (μM)	Comments
**std**	CGIGAVLKVLTTGLPALISWIKRKRQQ	0.8 ± 0.2 (*n* =25)	2.6 ± 0.6 (*n* = 25)	Melittin
**1**	CYGRKKRRQRR	>20	>20	Tat
**2**	CRQIKIWFQNRRMKWKK	>20	>20	Penetratin
**3**	CGLFEAIAGFIENGWEGMIDGWYG	>20	>20	HA2
**4**	CGLFEAIEGFIENGWEGMIDGWYG	16	>20	INF7
**5**	CGLFEAIAGFIENGWEGMIDGWYGYGRKKRRQRR	2.3 ± 0.5 (*n* = 10)	5.7 ± 1.1 (*n* = 10)	HA2-Tat
**6**	CGLFEAIEGFIENGWEGMIDGWYGYGRKKRRQRR	1.4 ± 0.4 (*n* = 10)	4.6 ± 1.2 (*n* = 10)	INF7-Tat
**7**	CGLFHAIAHFIHGGWHGLIHGWYGYGRKKRRQRR	5.9	0.3	H5WYG-Tat
**8**	CGLFKAIAKFIKGGWKGLIKGWYGYGRKKRRQRR	1.9	0.9	K5WYG-Tat
**9**	CGLFEAIAEFIEGGWEGLIEGWYGYGRKKRRQRR	>20	2.2	E5WYG-Tat
**10**	CGLFEAIAGFIENGWEGMIDGWYGRQIKIWFQNRRMKWKKGG	>20	>20	HA2-Penetratin
**11**	CGLFEAIEGFIENGWEGMIDGWYGRQIKIWFQNRRMKWKKGG	>20	>20	INF7-Penetratin

All peptides were >95% purity by HPLC analysis and demonstrated the correct HRMS profiles. Peptides were tested three times at each pH unless otherwise noted, and the values are reported as the mean. Standard Deviations are reported for peptides with larger *n* values to provide a reference for the reproducibility of the data. As solubility allowed, testing at pH 7.4 was pushed to the highest possible concentrations to determine as close as possible approximate IC_50_.

**Table 2:** Modifications to the INF7 portion of the chimeric peptide.

Number	Sequence	RBC Lysis IC_50_, pH 5.5 (μM)	RBC Lysis IC_50_, pH 7.4 (μM)
**12a**	CRLFEAIEGFIENGWEGMIDGWYGYGRKKRRQRR	0.8	3.5
**12b**	CELFEAIEGFIENGWEGMIDGWYGYGRKKRRQRR	1.5	>20
**12c**	CGGGLFEAIEGFIENGWEGMIDGWYGYGRKKRRQRR	1	0.9
**12d**	C(Nle)LFEAIEGFIENGWEGMIDGWYGYGRKKRRQRR	0.8	2.4
**12e**	CVLFEAIEGFIENGWEGMIDGWYGYGRKKRRQRR	0.72	>20
**12f**	C(b-Ala)LFEAIEGFIENGWEGMIDGWYGYGRKKRRQRR	0.38 ± 0.17 (*n* = 5)	>20 (*n* = 5)
**12g**	CSLFEAIEGFIENGWEGMIDGWYGYGRKKRRQRR	0.66	>20
**13a**	CGFFEAIEGFIENGWEGMIDGWYGYGRKKRRQRR	0.35	1.16
**13b**	CGKFEAIEGFIENGWEGMIDGWYGYGRKKRRQRR	>20	>20
**13c**	CGEFEAIEGFIENGWEGMIDGWYGYGRKKRRQRR	>20	>20
**13d**	CGGFEAIEGFIENGWEGMIDGWYGYGRKKRRQRR	>10	>10
**13e**	CGNFEAIEGFIENGWEGMIDGWYGYGRKKRRQRR	>10	>10
**14a**	CGLNEAIEGFIENGWEGMIDGWYGYGRKKRRQRR	>100	>100
**14b**	CGLGEAIEGFIENGWEGMIDGWYGYGRKKRRQRR	>20	>20
**14c**	CGLKEAIEGFIENGWEGMIDGWYGYGRKKRRQRR	>10	>10
**14d**	CGLEEAIEGFIENGWEGMIDGWYGYGRKKRRQRR	2.5	>10
**14e**	CGLAEAIEGFIENGWEGMIDGWYGYGRKKRRQRR	1.3 (max. 65%)	>10
**14f**	CGLLEAIEGFIENGWEGMIDGWYGYGRKKRRQRR	0.93	3.67
**15a**	CGLFGAIEGFIENGWEGMIDGWYGYGRKKRRQRR	0.4	1
**15b**	CGLFAAIEGFIENGWEGMIDGWYGYGRKKRRQRR	1.26	0.75
**15c**	CGLFNAIEGFIENGWEGMIDGWYGYGRKKRRQRR	1.12	1.78
**15d**	CGLFLAIEGFIENGWEGMIDGWYGYGRKKRRQRR	>10	>10
**15e**	CGLFKAIEGFIENGWEGMIDGWYGYGRKKRRQRR	0.71	1.27
**16a**	CGLFENIEGFIENGWEGMIDGWYGYGRKKRRQRR	>10	>10
**16b**	CGLFELIEGFIENGWEGMIDGWYGYGRKKRRQRR	0.23	1.93
**16c**	CGLFEEIEGFIENGWEGMIDGWYGYGRKKRRQRR	0.26	>10
**16d**	CGLFEKIEGFIENGWEGMIDGWYGYGRKKRRQRR	0.04	1.11
**17a**	CGLFEAKEGFIENGWEGMIDGWYGYGRKKRRQRR	>10	>10
**17b**	CGLFEAEEGFIENGWEGMIDGWYGYGRKKRRQRR	4.74	>10
**17c**	CGLFEALEGFIENGWEGMIDGWYGYGRKKRRQRR	0.8	5
**18a**	CGLFEAIWGFIENGWEGMIDGWYGYGRKKRRQRR	0.9	1.2
**18b**	CGLFEAIKGFIENGWEGMIDGWYGYGRKKRRQRR	2.2	6.5
**18c**	CGLFEAIHGFIENGWEGMIDGWYGYGRKKRRQRR	7.7	>80
**18d**	CGLFEAIRGFIENGWEGMIDGWYGYGRKKRRQRR	1.2	2.8
**18e**	CGLFEAIDGFIENGWEGMIDGWYGYGRKKRRQRR	3.7	>100
**18f**	CGLFEAIGGFIENGWEGMIDGWYGYGRKKRRQRR	2.1	2.1
**19a**	CGLFEAIENFIENGWEGMIDGWYGYGRKKRRQRR	2.7	3.09
**19b**	CGLFEAIEKFIENGWEGMIDGWYGYGRKKRRQRR	2.32	>10
**19c**	CGLFEAIEEFIENGWEGMIDGWYGYGRKKRRQRR	0.41	>10
**19d**	CGLFEAIEGGIENGWEGMIDGWYGYGRKKRRQRR	2.52	>10
**20a**	CGLFEAIEGAIENGWEGMIDGWYGYGRKKRRQRR	0.88	4.71
**20b**	CGLFEAIEGLIENGWEGMIDGWYGYGRKKRRQRR	0.24	1.97
**20c**	CGLFEAIEGGIENGWEGMIDGWYGYGRKKRRQRR	2.52	>10
**20d**	CGLFEAIEGAIENGWEGMIDGWYGYGRKKRRQRR	0.88	4.71
**20e**	CGLFEAIEGLIENGWEGMIDGWYGYGRKKRRQRR	0.24	1.97
**21a**	CGLFEAIEGFGENGWEGMIDGWYGYGRKKRRQRR	>10	>10
**21b**	CGLFEAIEGFAENGWEGMIDGWYGYGRKKRRQRR	1.59	>10
**21c**	CGLFEAIEGFLENGWEGMIDGWYGYGRKKRRQRR	0.59	2.8
**21d**	CGLFEAIEGFWENGWEGMIDGWYGYGRKKRRQRR	0.35	>10
**21e**	CGLFEAIEGFKENGWEGMIDGWYGYGRKKRRQRR	>10	>10
**21f**	CGLFEAIEGFNENGWEGMIDGWYGYGRKKRRQRR	>100	>100
**22a**	CGLFEAIEGFILNGWEGMIDGWYGYGRKKRRQRR	1.06	1.1
**22b**	CGLFEAIEGFIWNGWEGMIDGWYGYGRKKRRQRR	0.44	0.95
**22c**	CGLFEAIEGFIKNGWEGMIDGWYGYGRKKRRQRR	4.27	>10
**22d**	CGLFEAIEGFIGNGWEGMIDGWYGYGRKKRRQRR	6.16	>10
**23a**	CGLFEAIEGFIEPGWEGMIDGWYGYGRKKRRQRR	>10	>10
**23b**	CGLFEAIEGFIEPGWEGMIDGWYGYGRKKRRQRR	>10	>10
**23c**	CGLFEAIEGFIEGGWEGMIDGWYGYGRKKRRQRR	1.11	6.55
**23d**	CGLFEAIEGFIELGWEGMIDGWYGYGRKKRRQRR	1.34	1.31
**23e**	CGLFEAIEGFIEWGWEGMIDGWYGYGRKKRRQRR	0.54	5.18
**23f**	CGLFEAIEGFIEKGWEGMIDGWYGYGRKKRRQRR	1.52	>10
**24a**	CGLFEAIEGFIENEWEGMIDGWYGYGRKKRRQRR	max. 45% @ 2.0 uM	>100
**24b**	CGLFEAIEGFIENKWEGMIDGWYGYGRKKRRQRR	>10	>10
**24c**	CGLFEAIEGFIENNWEGMIDGWYGYGRKKRRQRR	6.39	>10
**24d**	CGLFEAIEGFIENHWEGMIDGWYGYGRKKRRQRR	3.06	2.9
**24e**	CGLFEAIEGFIENPWEGMIDGWYGYGRKKRRQRR	1.71	4.72
**24f**	CGLFEAIEGFIENAWEGMIDGWYGYGRKKRRQRR	1.88	1.28
**24g**	CGLFEAIEGFIENWWEGMIDGWYGYGRKKRRQRR	1.1	>10
**25a**	CGLFEAIEGFIENGEEGMIDGWYGYGRKKRRQRR	1.55	0.31
**24b**	CGLFEAIEGFIENGKEGMIDGWYGYGRKKRRQRR	>10	>10
**25c**	CGLFEAIEGFIENGLEGMIDGWYGYGRKKRRQRR	0.66	4.43
**26a**	CGLFEAIEGFIENGWDGMIDGWYGYGRKKRRQRR	0.55	1.3
**26b**	CGLFEAIEGFIENGWRGMIDGWYGYGRKKRRQRR	0.19	3.3
**26c**	CGLFEAIEGFIENGWKGMIDGWYGYGRKKRRQRR	0.34	>10
**26d**	CGLFEAIEGFIENGWHGMIDGWYGYGRKKRRQRR	2.54	>10
**26e**	CGLFEAIEGFIENGWNGMIDGWYGYGRKKRRQRR	1.47	>10
**26f**	CGLFEAIEGFIENGWLGMIDGWYGYGRKKRRQRR	0.84	2.03
**26g**	CGLFEAIEGFIENGWAGMIDGWYGYGRKKRRQRR	1.3	>10
**26h**	CGLFEAIEGFIENGWGGMIDGWYGYGRKKRRQRR	3.1	>10
**27a**	CGLFEAIEGFIENGWEAMIDGWYGYGRKKRRQRR	0.12(max. 65%)	0.95
**27b**	CGLFEAIEGFIENGWENMIDGWYGYGRKKRRQRR	0.42	>10
**27c**	CGLFEAIEGFIENGWEKMIDGWYGYGRKKRRQRR	1.01	8.99
**27d**	CGLFEAIEGFIENGWEEMIDGWYGYGRKKRRQRR	3.76	>10
**28a**	CGLFEAIEGFIENGWEG(M sulfoxide)IDGWYGYGRKKRRQRR	>100	>100
**28b**	CGLFEAIEGFIENGWEG(M sulfone)IDGWYGYGRKKRRQRR	>100	>100
**28c**	CGLFEAIEGFIENGWEGNIDGWYGYGRKKRRQRR	>10	>10
**28d**	CGLFEAIEGFIENGWEGQIDGWYGYGRKKRRQRR	>10	>10
**28e**	CGLFEAIEGFIENGWEGTIDGWYGYGRKKRRQRR	>10	>10
**28f**	CGLFEAIEGFIENGWEGSIDGWYGYGRKKRRQRR	>10	>10
**28g**	CGLFEAIEGFIENGWEGLIDGWYGYGRKKRRQRR	0.71	2.9
**28h**	CGLFEAIEGFIENGWEG(Nle)IDGWYGYGRKKRRQRR	0.53	3.94
**28i**	CGLFEAIEGFIENGWEGYIDGWYGYGRKKRRQRR	2.56	>10
**28j**	CGLFEAIEGFIENGWEGFIDGWYGYGRKKRRQRR	0.52	9.65
**28k**	CGLFEAIEGFIENGWEGIDGWYGYGRKKRRQRR (delete)	50	>100
**29a**	CGLFEAIEGFIENGWEGMLDGWYGYGRKKRRQRR	10.2	>10
**29b**	CGLFEAIEGFIENGWEGMADGWYGYGRKKRRQRR	>10	>10
**29c**	CGLFEAIEGFIENGWEGMGDGWYGYGRKKRRQRR	2.53	>10
**29d**	CGLFEAIEGFIENGWEGMNDGWYGYGRKKRRQRR	1.04	>10
**29e**	CGLFEAIEGFIENGWEGMKDGWYGYGRKKRRQRR	1.17	9.1
**29f**	CGLFEAIEGFIENGWEGMEDGWYGYGRKKRRQRR	>10	>10
**30a**	CGLFEAIEGFIENGWEGMIEGWYGYGRKKRRQRR	4.4	>10
**30b**	CGLFEAIEGFIENGWEGMIAGWYGYGRKKRRQRR	>10	2.83
**30c**	CGLFEAIEGFIENGWEGMIWGWYGYGRKKRRQRR	>10	>10
**30d**	CGLFEAIEGFIENGWEGMILGWYGYGRKKRRQRR	>10	>10
**31a**	CGLFEAIEGFIENGWEGMIDAWYGYGRKKRRQRR	1.4	>10
**31b**	CGLFEAIEGFIENGWEGMIDLWYGYGRKKRRQRR	0.32	0.96
**31c**	CGLFEAIEGFIENGWEGMIDIWYGYGRKKRRQRR	1.68	>10
**31d**	CGLFEAIEGFIENGWEGMIDWWYGYGRKKRRQRR	0.55 ± 0.25 (*n* = 8)	7.14 ± 0.73 (*n* = 8)
**31e**	CGLFEAIEGFIENGWEGMIDNWYGYGRKKRRQRR	6.07	7.26
**31f**	CGLFEAIEGFIENGWEGMIDKWYGYGRKKRRQRR	2.86	>10
**31g**	CGLFEAIEGFIENGWEGMIDEWYGYGRKKRRQRR	1.5	>10
**32a**	CGLFEAIEGFIENGWEGMIDGYGRKKRRQRR	>10	>10
**32b**	CGLFEAIEGFIENGWEGMIDGGGGYGRKKRRQRR	>10	>10
**33a**	CGLFEAIEGFIENGWEGMIDGLYGYGRKKRRQRR	4.24	1.75
**33b**	CGLFEAIEGFIENGWEGMIDGAYGYGRKKRRQRR	4.07	>10
**33c**	CGLFEAIEGFIENGWEGMIDGGYGYGRKKRRQRR	>10	>10
**33d**	CGLFEAIEGFIENGWEGMIDGNYGYGRKKRRQRR	2.5	>10
**33e**	CGLFEAIEGFIENGWEGMIDGKYGYGRKKRRQRR	6.6	>10
**33f**	CGLFEAIEGFIENGWEGMIDGEYGYGRKKRRQRR	4.26	>10
**34a**	CGLFEAIEGFIENGWEGMIDGWAGYGRKKRRQRR	>10	>10
**34b**	CGLFEAIEGFIENGWEGMIDGWHGYGRKKRRQRR	5.04	>10
**34c**	CGLFEAIEGFIENGWEGMIDGWNGYGRKKRRQRR	0.13	1.72
**34d**	CGLFEAIEGFIENGWEGMIDGWEGYGRKKRRQRR	3.94	>10
**35a**	CGLFEAIEGFIENGWEGMIDGWYAYGRKKRRQRR	2	>10
**35b**	CGLFEAIEGFIENGWEGMIDGWYWYGRKKRRQRR	0.17 (max. 70%)	3.05
**35c**	CGLFEAIEGFIENGWEGMIDGWYEYGRKKRRQRR	1.04 (max. 60%)	>10

All peptides were >95% purity by HPLC analysis and demonstrated the correct HRMS profiles. Peptides were tested three times at each pH unless otherwise noted, and the values are reported as the mean. Standard Deviations are reported for peptides with larger *n* values to provide a reference for the reproducibility of the data. As solubility allowed, testing at pH 7.4 was pushed to the highest possible concentrations to determine as close as possible approximate IC_50_.

**Table 3:** Modifications to the spacer between the peptide chimera, the Tat potion of the chimeric peptide, and miscellaneous modifications.

Number	Sequence	RBC Lysis IC_50_, pH 5.5 (μM)	RBC Lysis IC_50_, pH 7.4 (μM)
**36a**	GLFEAIEGFIENGWEGMIDGWYGCYGRKKRRQRR	2.09	2.1
**36b**	CGLFEAIEGFIENGWEGMIDGWYGGGGYGRKKRRQRR	0.7	7.9
**36c**	CGLFEAIEGFIENGWEGMIDGWYG(Peg 3)YGRKKRRQRR	1.92	>10
**36d**	CGLFEAIEGFIENGWEGMIDGWYG(Peg 6)YGRKKRRQRR	1.22	>10
**36e**	CGLFEAIEGFIENGWEGMIDGWYG(Peg11)YGRKKRRQRR	2.4	>100
**36f**	CGLFEAIEGFIENGWEGMIDGWYG(Peg 27)YGRKKRRQRR	28	>100
**37a**	CGLFEAIEGFIENGWEGMIDGWYGYGKKKKKQKK	7.78	>100
**37b**	CGLFEAIEGFIENGWEGMIDGWYGYGHKKHHQHH	0.90 ± 0.19 (*n* = 8)	>100 (*n* = 8)
**37c**	CGLFEAIEGFIENGWEGMIDGWYGYGRKKRRQRRR	1.77	6.82
**37d**	CGLFEAIEGFIENGWEGMIDGWYGYGRKKRRQR	0.75	>100
**37e**	CGLFEAIEGFIENGWEGMIDGWYGYGRKKRRQ	0.96	>100
**37f**	CGLFEAIEGFIENGWEGMIDGWYGYGRKKRR	1.61	>100
**37g**	CGLFEAIEGFIENGWEGMIDGWYGYGRKKR	7.8	>100
**37h**	CGLFEAIEGFIENGWEGMIDGWYGYGHKKHHQHR	0.58 ± 0.17 (*n* = 10)	>100 (*n* = 10)
**38a**	GLFEAIEGFIENGWEGMIDGWYGYGRKKRRQRRC	0.96	>10
**38b**	YGRKKRRQRRGLFEAIEGFIENGWEGMIDGWYGC	2.91	>100
**38c**	CYGRKKRRQRRGLFEAIEGFIENGWEGMIDGWYG	1.17	>20
**38d**	rrqrrkkrgygywgdimgewgneifgeiaeflgc	5	>100
**38e**	crrqrrkkrgygywgdimgewgneifgeiaeflg	0.1	1.51
**38f**	cglfeaiegfiengwegmidgwygygrkkrrqrr	2.3	>20

All peptides were >95% purity by HPLC analysis and demonstrated the correct HRMS profiles. Peptides were tested three times at each pH unless otherwise noted, and the values are reported as the mean. Standard Deviations are reported for peptides with larger *n* values to provide a reference for the reproducibility of the data. As solubility allowed, testing at pH 7.4 was pushed to the highest possible concentrations to determine as close as possible approximate IC_50_.

**Table 4:** Stapled and lipidated peptide analogs.

Number	Sequence	RBC Lysis IC_50_, pH 5.5 (μM)	RBC Lysis IC_50_, pH 7.4 (μM)
**39a**	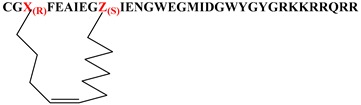	0.48	>10
**39b**	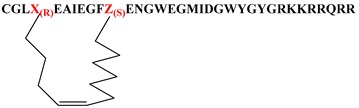	0.19	>10
**39c**	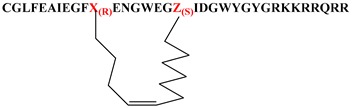	1.89	>10
**39d**	CGLFEAIEGFIENGWEGMIDGWYGYGRKKRRQRRK(Stearoyl)	0.58 ± 0.2 (*n* = 6)	0.94 ± 0.4 (*n* = 6)
**39e**	(stearoyl)GLFEAIEGFIENGWEGMIDGWYGYGRKKRRQRRC	0.7	>10

All peptides were >95% purity by HPLC analysis and demonstrated the correct HRMS profiles. Peptides were tested three times at each pH unless otherwise noted, and the values are reported as the mean. Standard Deviations are reported for peptides with larger *n* values to provide a reference for the reproducibility of the data. As solubility allowed, testing at pH 7.4 was pushed to the highest possible concentrations to determine as close as possible approximate IC_50_ values. For peptide **40a**, the stearoyl group was acylated onto the N-terminus of the peptide, and for peptide **40b**, a lysine was added to the C-terminus of the peptide and the epsilon amine was acylated with the steroyl group.

**Table 5:** Combination peptide analogs.

Number	Sequence	RBC Lysis IC_50_, pH 5.5 (μM)	RBC Lysis IC_50_, pH 7.4 (μM)
**40**	CELFEAIEGFIENGWEGLIDGWYGYGRKKRRQRR	2.12	>10
**41**	CGLFGAIEGFIENGWEGLIDGWYGYGRKKRRQRR	0.41	0.9
**42**	CGLFEAIEGFIENGWKGMIDWWYGYGRKKRRQRR	0.66	0.29
**43**	CGLFEAIEGFIENGWEGLIDAWYGYGRKKRRQRR	0.62	>10
**44**	CELFEAIWGFIENGWEGMIDGWYGGGGYGRKKRRQRR	1 (max.50%)	5 (max.60%)
**45**	CELFEAIEGFIENGWEGLIDGWYGYGRKKRRQRRR	2.98	4.32
**46**	CRLFEAIWGFIENGWEGMIDGWYGGGGYGRKKRRQRR	0.35	0.12
**47**	CGLFGAIEGFIENGWEGLIDGWYGYGRKKRRQRRR	1.55	3.97
**48**	CGLFEEIEGFIENGWEGLIDWWYGYGRKKRRQRR	0.72	>10
**49**	CELFGAIWGFIENGWEGLIDGWYGYGRKKRRQRR	>10	>10
**50**	CELFGAIEGFIENGWEGLIDGWYGYGRKKRRQRRR	2	max. 35% @ >10
**51**	CELFGAIEGFIENGWKGLIDWWYGYGRKKRRQRR	0.14	3.29
**52**	CGLFGAIEGFIENGWKGLIDAWYGYGRKKRRQRR	0.1	3.41
**53**	CGLFEAIEGFIENGLKGLIDAWYGYGRKKRRQRR	0.18	>10
**54**	CELFGAIEGFIENGWKGLIDWWYGYGRKKRRQRR	0.14	3.29
**55**	CELFGAIEGFIENGWKGLIDAWYGYGRKKRRQRR	0.71	3.19
**56**	CGIFGAIEGFIENGWWGLIDAWYGYGRKKRRQRR	0.07	2.35
**57**	CGFFEAIEGFIENGLKGLIDAWYGYGRKKRRQRR	0.17	3.99
**58**	CGLAEAIEGFIENGLKGLIDWWYGYGRKKRRQRR	0.06	2
**59**	CGLFEAIA**E**FIEGGWEGLIEGWYGYGRKKRRQRR	0.79	>10
**60**	C(b-Ala)GFEFIEEFIENGLKNLIDWWYGYGRKKRRQRR	1.14	12.8
**61**	CGLFGEIEELIEEGLKNLIDWWYGYGRKKRRQRR	0.97	8.07
**62**	CGLFGEIEELIEEGLKNLIDWWYGYGHKKHHQHR	1.28	>20
**63**	CGLFGEIEELIEEGLENLIDWWYGYGRKKRRQRR	3.6	>20
**64**	CGLFGEIEELIEEGLENLIDWWYGYGHKKHHQHR	3.91	>20
**65**	CGLFGEIEEFIEEGLENLIDWWYGYGRKKRRQRR	3.15	>20
**66**	CGLFGEIEEFIEEGWENFIDWWYGYGRKKRRQRR	4.08	>20
**67**	CGLFGEIEELIEEGWEGLIDWWYGYGRKKRRQRR	3.4	>20
**68**	CGLFEEIEELIEEGLENLIDWWYGYGRKKRRQRR	4.11	>20
**69**	CGLFEEIEELIEEGLENLIDWWYGYGHKKHHQHR	8.73	>20
**70**	CGLFEEIEELIEEGLKNLIDWWYGYGHKKHHQHR	2.28	>20

All peptides were >95% purity by HPLC analysis and demonstrated the correct HRMS profiles. Peptides were tested three times at each pH unless otherwise noted, and the values are reported as the mean. Standard Deviations are reported for peptides with larger *n* values to provide a reference for the reproducibility of the data. As solubility allowed, testing at pH 7.4 was pushed to the highest possible concentrations to determine as close as possible approximate IC_50_.

## References

[1] Fire A., Xu S., Montgomery M.K., Kostas S.A., Driver S.E., Mello C.C. (1998). Potent and specific genetic interference by double-stranded RNA in Caenorhabditis elegans. Nature.

[2] Stanton M.G., Colletti S.L. (2010). Medicinal Chemistry of siRNA Delivery. J. Med. Chem..

[3] EErazo-Oliveras A., Muthukrishnan N., Baker R., Wang T.Y., Pellois J.P. (2012). Improving the endosomal escape of cell-penetrating peptides and their cargos. Pharmaceuticals.

[4] Endoh T., Ohtsuki T. (2009). Cellular siRNA delivery using cell-penetrating peptides modified for endosomal escape. Adv. Drug Del. Rev..

[5] Weise K., Reed J. (2008). Fusion peptides and transmembrane domains of fusion proteins are characterized by different but specific structural properties. Chembiochem.

[6] Lundberg P., El-Andaloussi S., Sutlu T., Johansson H., Langel U. (2007). Delivery of short interfering RNA using endosomolytic cell-penetrating peptides. FASEB J..

[7] Avci F.G., Akbulut B.S., Ozkirimli E. (2018). Membrane active peptides and their biophysical characterization. Biomolecules.

[8] Liu Q., Zhao H., Jiang Y., Tian Y., Wang D., Lao Y., Xu N., Li Z. (2016). Development of a lytic peptide derived from BH3-only proteins. Cell Death Discov..

[9] Epand R.M. (2003). Fusion peptides and the mechanism of viral fusion. Biochim. Biophys. Acta.

[10] Wimley W.C. (2010). Describing the mechanism of antimicrobial peptide action with the interfacial activity model. ACS Chem. Bio..

[11] Dufourc E.J., Buchoux S., Toupé J., Sani M.A., Jean-François F., Khemtémourian L., Grélard A., Loudet-Courrèges C., Laguerre M., Elezgaray J. (2012). Membrane interacting peptides: from killers to helpers. Curr. Prot. Pept. Sci..

[12] Tamm L.K., Han X. (2000). Viral Fusion Peptides: A tool set to disrupt and connect biological membranes. Biosci. Rep..

[13] Guidotti G., Brambilla L., Rossi D. (2017). Cell-penetrating peptides: from basic research to clinics. Trends Pharmacol. Sci..

[14] Sugita T., Yoshikawa T., Mukai Y., Yamanda N., Imai S., Nagano K., Yoshida Y., Shibata H., Yoshiaok Y., Nakagawa S. (2007). Improved cytosolic translocation and tumor-killing activity of Tat-shepherdin conjugates mediated by co-treatment with Tat-fused endosome-disruptive HA2 peptide. Biochem. Biophys. Res. Comm..

[15] Lee Y., Jophnson G., Pellois J.P. (2012). Modeling of the Endosomolytic Activity of HA2-TAT Peptides with Red Blood Cells and Ghosts. Biochemistry.

[16] Angeles-Boza A.M., Erazo-Oliveras A., Lee Y.J., Pellois J.P. (2010). Generation of Endosomolytic Reagents by Branching of Cell-Penetrating Peptides: Tools for the Delivery of Bioactive Compounds to Live Cells in Cis or Trans. Bioconj. Chem..

[17] Lee Y., Johnson G., Peltier G.C., Pellois J.P. (2011). A HA2-Fusion tag limits the endosomal release of its protein cargo despite causing endosomal lysis. Biochim. Biophys. Acta.

[18] Liou J., Liu B.R., Martin A.L., Huang Y., Chiang H.-J., Lee H.-J. (2012). Protein transduction in human cells is enhanced by cell-penetrating peptides fused with an endosomolytic HA2 sequence. Peptides.

[19] Ye S.-F., Tian M., Wang T.-X., Ren L., Wang D., Shen L.-H., Shang T. (2012). Synergistic effects of cell-penetrating peptide Tat and fusogenic peptide HA2-enhanced cellular internalization and gene transduction of organosilica nanoparticles. Nanomed. Nanotech Bio. Med..

[20] Oliverira S., van Rooy I., Kranenburg O., Storm G., Schiffelers R.M. (2007). Fusogenic peptides enhance endosomal escape improving siRNA-induced silencing of oncogenes. Int. J. Pharm..

[B21-molecules-24-02079] 21.Thomas J. Tucker, Merck Research Laboratories, West Point, PA, USA. There are different Tat sequences that have been used in the literature for delivery; we chose the YG-Tat (YGRKKRRQRR) sequence for this particular study as unpublished internal data showed that this sequence exhibited the most consistent CPP behavior in multiple cell lines, and also in general showed very good physical properties and behavior, 2016.

[22] Miletti F. (2012). Cell-penetrating peptides: classes, origin, and current landscape. Drug Disc. Today.

[23] Mechtler K., Wagner E. (1997). Gene transfer mediated by influenza virus peptides: the role of peptide sequences. New J. Chem..

[24] Sung M.S., Mun J.Y., Kwon O., Oh D.B. (2013). Efficient myogenic differentiation of human adipose-derived stem cells by the transduction of engineered MyoD protein. Biochem. Biophys. Res. Comm..

[25] Lin N., Zheng W., Li L., Liu H., Wang T., Wang P., Ma X. (2014). A novel system enhancing the endosomal escapes of peptides promotes Bak BH3 peptide inducing apoptosis in lung cancer A549 cells. Targ. Oncol..

[26] Garcia-Sosa A.T., Tulp I., Langel K., Langel U. (2014). Peptide-ligand binding modeling of siRNA with cell penetrating peptides. Biomed. Res. Int..

[27] Friemann K., Arukuusk P., Kurrikoff K., Ferreira Vasconcelos L.D., Veiman K.-L., Uusna J., Margus H., Garcia-Sosa A.T., Pooga M., Langel U. (2016). Optimization of in vivo delivery with NickFect peptide vectors. J. Cont. Rel..

[28] Durell R.D., Martin I., Ruysschaert J.-M., Shai Y., Blumenthal R. (1997). What studies of fusion peptides tell us about viral envelope glycoprotein-mediated membrane fusion. Mol. Memb. Biol..

[29] Cross K.J., Wharton S.A., Skehel J.J., Wiley D.C., Steinhauser D.A. (2001). Studies on influenza haemagglutinin fusion peptide mutants generated by reverse genetics. EMBO J..

[30] Han X., Bushweller J.H., Cafiso D.S., Tamm L.K. (2001). Membrane structure and fusion-triggering conformational change of the fusion domain from influenza hemagglutinin. Nat. Struct. Bio..

[31] Lorieau J.L., Louis J.M., Bax A. (2010). The complete influenza hemagglutinin fusion domain adopts a tight helical hairpin arrangement at the lipid:water interface. Proc. Natl. Acad. Sci..

[32] Lorieau J.L., Louis J.M., Bax A. (2011). Helical Hairpin Structure of Influenza Hemagglutinin Fusion Peptide Stabilized by Charge−Dipole Interactions between the N-Terminal Amino Group and the Second Helix. J. Am. Chem. Soc..

[33] Midoux P., Kichler A., Boutin V., Maurizot J.-C., Monsigny M. (1998). Membrane Permeabilization and Efficient Gene Transfer by a Peptide Containing Several Histidines. Bioconj. Chem..

[34] Schafmeister C.E., Po J., Verdine G.L. (2000). An All-Hydrocarbon Cross-Linking System for Enhancing the Helicity and Metabolic Stability of Peptides. J. Am. Chem. Soc..

[35] Kutchukian P.S., Yang J.S., Verdine G.L., Shakhnovich E.I. (2009). An all-atom model for stabilization of α-helical structure in peptides by hydrocarbon staples. J. Am. Chem. Soc..

[36] Kim Y.-W., Grossmann T.N., Verdine G.L. (2011). Synthesis of all-hydrocarbon stapled α-helical peptides by ring-closing olefin metathesis. Nat. Protoc..

[37] Martin I., Teixido M., Giralt E. (2011). Design, synthesis and characterization of a new anionic cell-penetrating peptide: SAP(E). ChemBioChem.

[38] Sitaram N. (2006). Antimicrobial peptides with unusual amino acid compositions and unusual structures. Curr. Med. Chem..

[39] Harris F., Dennison S.R., Phoenix D.A. (2011). Anionic antimicrobial peptides from eukaryotic organisms and their mechanisms of action. Curr. Chem. Bio..

[40] Henry S.M., El-Sayed M.E., Pirie C.M., Hoffman A.S., Stayton P.S. (2006). pH-responsive poly (styrene-alt-maleic anhydride) alkylamide copolymers for intracellular drug delivery. Biomacromolecules.

